# Propensity score analysis of stented versus rapid deployment aortic bioprostheses in patients with small aortic annulus

**DOI:** 10.1093/icvts/ivaf241

**Published:** 2025-10-10

**Authors:** Giorgia Cibin, Augusto D'Onofrio, Valentina Lombardi, Emma Bergonzoni, Giulia Lorenzoni, Elisa Gastino, Giuseppe Evangelista, Enrico Giuseppe Italiano, Irene Cao, Dario Gregori, Chiara Tessari, Gino Gerosa

**Affiliations:** Division of Cardiac Surgery, Department of Cardiac, Thoracic, Vascular Sciences and Public Health, University of Padova, Padova 35128, Italy; Division of Cardiac Surgery, University of Roma “Tor Vergata”, Rome 00133, Italy; Division of Cardiac Surgery, University of Roma “Tor Vergata”, Rome 00133, Italy; Division of Cardiac Surgery, Department of Cardiac, Thoracic, Vascular Sciences and Public Health, University of Padova, Padova 35128, Italy; Unit of Biostatistics, Epidemiology and Public Health, Department of Cardiac, Thoracic, Vascular Sciences and Public Health, University of Padova, Padova 35128, Italy; Division of Cardiac Surgery, IRCCS Policlinico San Donato, San Donato Milanese, Milano 20097, Italy; Division of Cardiac Surgery, Department of Cardiac, Thoracic, Vascular Sciences and Public Health, University of Padova, Padova 35128, Italy; Division of Cardiac Surgery, Department of Cardiac, Thoracic, Vascular Sciences and Public Health, University of Padova, Padova 35128, Italy; Division of Cardiac Surgery, Department of Cardiac, Thoracic, Vascular Sciences and Public Health, University of Padova, Padova 35128, Italy; Unit of Biostatistics, Epidemiology and Public Health, Department of Cardiac, Thoracic, Vascular Sciences and Public Health, University of Padova, Padova 35128, Italy; Division of Cardiac Surgery, Department of Cardiac, Thoracic, Vascular Sciences and Public Health, University of Padova, Padova 35128, Italy; Division of Cardiac Surgery, Department of Cardiac, Thoracic, Vascular Sciences and Public Health, University of Padova, Padova 35128, Italy

**Keywords:** surgical aortic valve replacement, rapid-deployment valve, stented aortic valve, small anulus

## Abstract

**Objectives:**

Haemodynamic studies have demonstrated the excellent performance of rapid deployment (RD) valves. This retrospective single-centre study aimed to compare early and medium-term outcomes of RD bioprostheses versus conventional stented valves in patients with small aortic annuli.

**Methods:**

We included patients who underwent isolated or combined surgical aortic valve replacement (SAVR) with Magna Ease (ME) and Intuity (Edwards Lifesciences, Irvine, CA) sizes 19 and 21 at our institution between June 2016 and March 2022. Follow-up was conducted through scheduled visits and echocardiograms at the study site, or via telephonic interviews with patients and/or referring cardiologists. A propensity score weighting analysis was performed to account for baseline differences between the 2 cohorts.

**Results:**

A total of 666 consecutive patients underwent SAVR with the 2 devices. ME was implanted in 367 patients (55.1%) and Intuity in 299 (44.9%). ME size 19 or 21 was used in 105 patients (35.1%), and Intuity size 19 or 21 in 115 patients (31.3%). Our study population comprised 220 patients. There were no significant differences in postoperative complications. Intuity demonstrated significantly lower gradients overall (mean gradients: 12 mmHg vs 16 mmHg, *P* < 0.001) and for size 21 (mean gradients: 12 mmHg vs 15 mmHg, *P* < 0.001). Mid-term survival and rehospitalization rates were similar between the 2 devices (5-year rehospitalization rate: 17% ME vs 20.9% Intuity, *P* = 0.57; 5-year survival: 81.9% ME vs 88% Intuity, *P* = 0.761).

**Conclusions:**

In patients with small aortic annuli, RD bioprostheses provide superior haemodynamic outcomes compared to conventional stented valves. However, perioperative outcomes, mid-term survival, and rehospitalization rates are similar between the 2 devices.

## Graphical abstract

**Figure:**
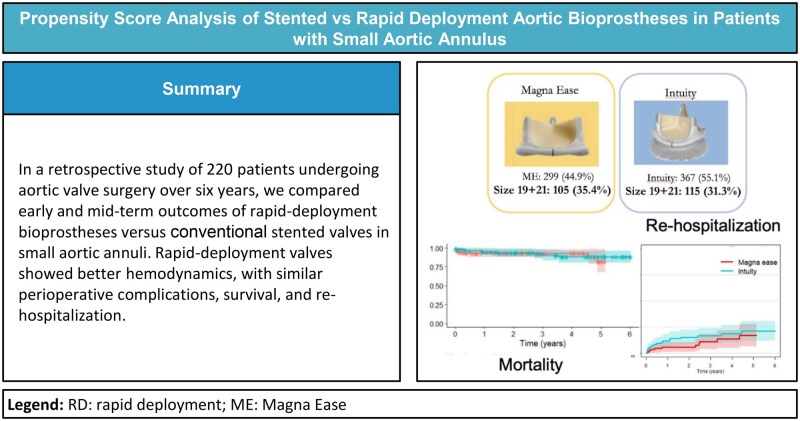

## INTRODUCTION

Patients with small aortic annulus face higher risk of patient-prosthesis mismatch (PPM) which worsens clinical and haemodynamic outcomes. Surgical aortic valve replacement (SAVR) using small bioprostheses is a standard option, but it often increases PPM resulting in higher transvalvular gradients, reduced survival for structural valve deterioration (SVD).^1^ Aortic annular enlargement can decrease PPM incidence but adds surgical risk and longer surgical times.^2^

Rapid deployment (RD) bioprostheses were developed to simplify minimally invasive surgery and shorten operative times.^3^ These valves help enlarge the left ventricular outflow tract, reduce transvalvular gradients, increase effective orifice area, and lower PPM rates.^4^ The aim of this retrospective single-centre study was to compare early and medium-term clinical and haemodynamic outcomes of conventional stented valves versus RD valves in patients with small aortic annulus.

## METHODS

All consecutive patients who underwent isolated or combined SAVR for aortic stenosis with stented Magna Ease (ME) and RD Intuity (Edwards Lifesciences, Irvine, CA) bioprostheses at our institution between June 2016 and March 2022 were included. Combined procedures involved the mitral valve, aorta, or coronary arteries. Following literature criteria, and in the absence of a clearly cut-off, a small annulus was defined as accommodating a prosthesis ≤21 mm based on intraoperative sizing.^5^^,^^6^ It must be acknowledged that the largest prosthesis cannot always be implanted, as anatomical factors such as severe root calcification may require smaller devices. Exclusion criteria were aortic regurgitation, endocarditis, dissection, and annular enlargement. Informed consent for data collection and subsequent analysis was obtained from all patients at the time of hospital admission, both verbally and through a signed consent form. The data were then anonymized and entered an institutional database. The institutional valvular registry (PRISMA study) was authorized by the ethics committee (5973/AO/24). Implant choice, ME or Intuity, depended on surgeon’s preference, intraoperative findings, and experience. Procedures were performed under general anaesthesia through full sternotomy, mini-sternotomy (J or inverted T), or mini-thoracotomy (second intercostal space). Intuity bioprostheses were implanted following standard technique.^7^ Sizing was performed intraoperatively using the same sizer for both stented and RD bioprostheses; no pre-operative angio-CT was performed. Follow-up included scheduled visits and echo at study site. Alternatively, telephonic interviews with patients and/or referring cardiologists were performed. Haemodynamic outcomes included mean and peak transvalvular gradients and Aortic Valve Area indexed (AVAi). Clinical outcomes included all-cause and cardiovascular mortality, major bleeding, stroke, acute kidney injury requiring continuous veno-venous hemofiltration (CVVH), permanent pacemaker implantation (PPI), and PPM. Preoperative variables followed European system for cardiac operative risk evaluation (EuroSCORE) definitions^8^ and postoperative outcomes adhered to updated Valve Academic Research Consortium (VARC) definitions.^9^ Re-hospitalization was defined as any unplanned admission after discharge for cardiovascular causes, including arrhythmias, early SVD, episodes of heart failure, or wound-related complications.

### Study devices

The Carpentier-Edwards Perimount Magna Ease is a cobalt-chromium stented bioprosthesis with 3 bovine pericardial leaflets treated using the Thermafix process. Implanted in the supra-annular position, it is suitable for both aortic stenosis and regurgitation. The Intuity bioprosthesis, and its updated Intuity Elite version, shares the ME’s design, including Thermafix-treated pericardial leaflets, but adds a balloon-expandable subannular skirt frame inspired by transcatheter bioprostheses, serving both as anchor and seal. Implantation technique has been detailed elsewhere.^10^^,^^11^ Briefly, after native leaflets removal and annular decalcification, 3 guiding sutures are positioned at the nadir of each sinus. The bioprosthesis is deployed into the annulus, the balloon is inflated, and the delivery system is removed before tying the sutures. The Intuity bioprosthesis is specifically indicated for aortic stenosis and contraindicated in aortic regurgitation and endocarditis.

### Statistical analysis

Descriptive statistics were reported as median (I_III quartiles) for continuous variables and absolute numbers (percentages) for categorical variables. Continuous variables were compared using the Wilcoxon-Kruskal-Wallis, while categorical variables were analysed with Pearson’s Chi-squared or Fisher’s exact test. A propensity score weighting approach was used to account for potential confounding related to the non-random allocation of patients. Propensity score weighting uses the estimated propensity score to assign a weight to each individual in the sample, generating a weighted population in which the distribution of baseline covariates is balanced across treatment groups. Propensity scores were estimated using covariate balancing propensity score (CBPS),^12^ with trimming at the 90° quartile. Complete case analysis was performed.

Variables included in the estimation were age, gender, body surface area (BSA), hypertension, dyslipidaemia, diabetes, extracardiac arteriopathy, previous cardiac surgery, neurological dysfunction, renal function, pre-operatory cardiac rhythm, STS score, and pre-operatory left ventricle function. Covariate balance was evaluated using standardized mean differences (SMDs) which provide a sample size-independent measure of comparability. In accordance with best practice, descriptive statistics for the weighted pseudopopulation and *P*-values were not reported, as they do not reliably reflect covariate distribution balance.^13–15^ After weighting, all covariates were balanced (**Figure S1**), except gender, which was included as an adjustment variable in outcome models. Weighted logistic regression was adopted for binary outcomes, reported as odds ratio (OR), 95% confidence interval (CI), and *P*-value. A weighted Gamma model was employed for continuous outcomes, ie, haemodynamic parameters, given the non-normal distribution of all the continuous outcomes considered. The marginal effect was computed considering the partial derivatives of the marginal expectation. Results were expressed as average marginal effects (AMEs), with 95% CI and *P*-value. Models for postoperative haemodynamic parameters were adjusted for baseline values.

Inverse probability of treatment weighting (IPTW) was preferred over matching to retain the entire cohort, preserving statistical power and avoiding loss of information from excluded patients. This is particularly relevant in the current context, where reducing the sample size would substantially limit the precision of the estimates. Compared to full covariate adjustment, IPTW provides a parsimonious approach reducing risks of overfitting and residual confounding when covariate distributions differ. By summarizing confounding into a single score and generating a weighted pseudo-population, IPTW improves balance and interpretability. Time-to-event outcomes were analysed with survival and readmission distributions. Survival was assessed using the Kaplan-Meier method, while cumulative incidence functions were used for readmission, accounting for competing risks. Weighted Cox proportional hazards models incorporating propensity score-derived weights were applied, and results expressed as hazard ratios (HRs) with 95% CI and *P*-values. Analyses were performed using R software^16^ within the packages rms, CBPS^17^ and WeightIt^16^ for propensity score weighting procedure estimation, and margins^18^ for AME computation.

## RESULTS

### Study population

**Figure 1.**
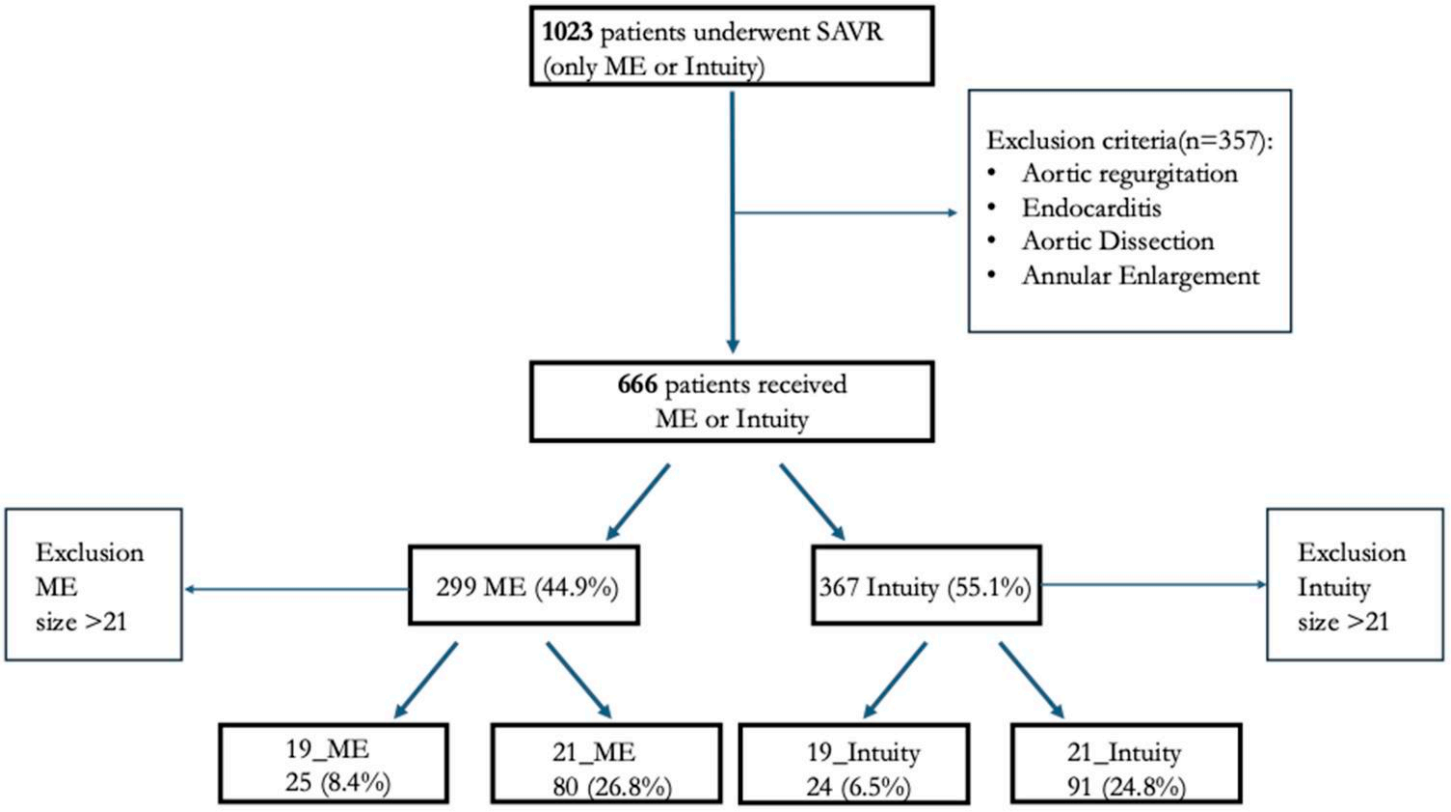
Consort Diagram Representing Our Study Population

**Figure 1** shows our study population. **Table 1** reports baseline and preoperative echocardiographic characteristics. A total of 666 patients underwent valve replacement with either ME or Intuity prostheses: 299 (44.9%) received ME and 367 (55.1%) Intuity. Only patients implanted with 19- or 21-mm valves were considered, resulting in a study cohort of 220 patients. Specifically, ME included 25 (8.4%) of 19-mm and 80 (26.8%) of 21-mm, while Intuity included 24 (6.5%) of 19-mm and 91 (24.8%) of 21-mm. After propensity adjustment, significant differences persisted between groups. Female gender was more prevalent in the Intuity cohort (79% vs 63%; *P* = .008). Intuity patients were more symptomatic (NYHA3: 73% vs 43%; *P* < .001), had lower estimated glomerular filtration rate (eGFR) (66 [IQR = 52-81] vs 78 [IQR = 64-89] mg/dl; *P* = .008), and showed higher STS mortality score (1.97% [IQR = 1.57-2.63] vs 1.56% [IQR = 1.07-2.37]; *P* = .008). EuroSCORE II was also higher, though not significant. Preoperative echocardiographic variables remained similar.

**Table 1.** Pre-Operative Characteristics

Variable	Total (n = 220)	Magna Ease (n = 105)	Intuity (n = 115)	*P*-value
Age (y)	73 (69-78)	73 (68-78)	74 (69-78)	.500
Female gender	157 (71%)	66 (63%)	91 (79%)	.008
Body surface area (m^2^)	1.72 (1.62-1.86)	1.78 (1.62-1.89)	1.70 (1.62-1.81)	.120
Body mass index	25.8 (23.1-29.1)	25.8 (22.5-29.4)	25.7 (23.3-28.8)	.900
Arterial hypertension	187 (85%)	85 (81%)	102 (89%)	.110
Diabetes mellitus	57 (26%)	29 (28%)	27 (23%)	.500
Insulin therapy	11 (5%)	5 (4.9%)	6 (5.2%)	.600
NYHA functional class				<.001
I	17 (7.7%)	14 (13%)	3 (2.6%)	
II	74 (33.6%)	46 (44%)	28 (24.3%)	
III+IV	129 (58.7%)	45 (43%)	84 (73.1%)	
Peripheral arterial disease	47 (21%)	17 (16%)	30 (26%)	.074
COPD	13 (5.9%)	9 (8.6%)	4 (3.5%)	.110
Neurological dysfunction	10 (4.5%)	5 (4.8%)	5 (4.3%)	>.900
Creatinine (mg/dl)	0.85 (0.74-1.04)	0.86 (0.75-1.04)	0.85 (0.72-1.04)	.800
GFR (mL/min/1.73 m^2^)	71 (56-84)	78 (64-89)	66 (52-81)	.008
Dialysis	1 (0.5%)	0 (0%)	1 (0.9%)	>.900
Haemoglobin (g/dl)	12.6 (11.7-13.6)	13 (11.9-13.8)	12.5 (11.5-13.4)	.017
Cardiac rhythm				.300
Sinus rhythm	191 (86.8%)	87 (82.8%)	104 (90.4%)	
Permanent AF	16 (7.3%)	11 (10.5%)	5 (4.4%)	
Paroxysmal AF	6 (2.7%)	3 (2.9%)	3 (2.6%)	
Pacemaker	7 (3.2%)	4 (3.8%)	3 (2.6%)	
Previous AMI				.026
No	209 (95%)	102 (98%)	107 (93%)	
< 90 days	13 (4.1%)	1 (1%)	8 (7%)	
Coronary artery disease	65 (29.5%)	41 (39%)	24 (20.9%)	.130
Previous cardiac surgery	12 (5.5%)	7 (6.7%)	5 (4.3%)	.400
EuroSCORE II	1.65 (1.12-2.76)	1.56 (1.08-2.62)	1.82 (1.21-2.87)	.120
STS mortality score	1.80 (1.21-2.54)	1.56 (1.07-2.37)	1.97 (1.57-2.63)	.008
Peak aortic gradient (mmHg)	78 (65-90)	76 (63-90)	78 (66-90)	.600
Mean aortic gradient (mmHg)	48 (40-57)	48 (39-57)	46 (41-56)	.600
AVAi (cm^2^/m^2^)	0.46 (0.38-0.55)	0.46 (0.4-0.58)	0.47 (0.37-0.52)	.500
LVEF (%)	60 (55-66)	60 (55-66)	61 (56-66)	.085

Continuous data are median (I quartile-III quartile), categorical data are absolute numbers (percentages).

Abbreviations: AF, atrial fibrillation; AMI, acute myocardial infarction; AVAi, aortic valve area indexed; COPD, chronic obstructive pulmonary disease; EuroSCORE, European System for Cardiac Operative Risk Evaluation; GFR, glomerular filtration rate; LVEF, left ventricular ejection fraction; NYHA, New York Heart Association; STS, Society of Thoracic Surgeons.

### Surgical procedure

**Table 2** summarizes intraoperative variables. Intuity were more frequently implanted through minimally invasive approaches compared to ME (16.5% vs 1.9%; *P* < .001) and were more often used in isolated SAVR (59.1% vs 56.2%; *P* = .001). Surgical times were significantly shorter with Intuity: cardiopulmonary bypass (isolated SAVR) averaged 88 min [IQR = 69-113] vs 109 min [IQR = 96-120] for ME (*P* < .001), while cross-clamp time was 66 min [IQR = 48-82] vs 88 min [IQR = 79-96] (*P* < .001). All ME prostheses were implanted supra-annularly.

**Table 2.** Procedural Characteristics (Data Are Absolute Numbers-Percentages) and Distribution of Aortic Cross-Clamp Time and Cardiopulmonary Bypass Time according to Valve Type in the Study Population Overall, and in Isolated and Combined Procedures (Data Are Median-I Quartile/III Quartile)

Variable	Total (n = 220)	Magna Ease (n = 105)	Intuity (n = 115)	*P*-value
**Surgical approach**				<.001
Full sternotomy	199 (90.4%)	103 (98.1%)	96 (83.5%)	
Ministernotomy	18 (8.2%)	2 (1.9%)	16 (13.9%)	
Minithoracotomy	3 (1.4%)	0 (0%)	3 (2.6%)	
**Surgical procedure**				.001
Isolated SAVR	124 (56.4%)	56 (56.2%)	68 (59.1%)	
Combined procedure	96 (43.6%)	49 (43.8%)	47 (40.9%)	
**Aortic cross-clamp time (min)**				
Isolated SAVR	79 (61-94)	88 (79-96)	66 (48-82)	<.001
Combined procedure	107 (84-140)	137 (105-158)	90 (70-110)	<.001
Overall	89 (68-111)	100 (86-134)	74 (54-98)	<.001
**Cardiopulmonary bypass time (min)**				
Isolated SAVR	101 (81-120)	109 (96-120)	88 (69-113)	<.001
Combined procedure	140 (120-186)	178 (142-200)	122 (97-139)	<.001
Overall	114 (90-148)	132 (109-180)	104 (76-129)	<.001

Combined procedure are myocardial revascularization, ascending aorta substitution, and mitral valve repair or replacement.

Abbreviation: SAVR, surgical aortic valve replacement.

### Postoperative outcomes

Postoperative clinical outcomes are shown in **Table 3** and echocardiographic parameters in **Table 4**. Perioperative outcomes were comparable between groups. Permanent pacemaker implantation (PPI) occurred in 8 Intuity patients (7%) and 7 ME patients (6.7%) without significant difference (OR = 1.02; 95% CI, 0.47-2.21). VARC-2 all-cause mortality was 3.2% overall, with similar rates between groups (Intuity 1.7% vs ME 4.8%; OR = 0.62; 95% CI, 0.20-1.89).

**Table 3.** Distribution of Postoperative Variables according to Valve Type in the Study Population Overall and in the 2 Groups of Valves; Categorical Data Are Absolute Numbers (Percentages)

Variable	Total (n = 220)	Magna Ease (n = 105)	Intuity (n = 115)	Odds ratio	95% CI
VARC-2 all-cause mortality	7 (3.2%)	5 (4.8%)	2 (1.7%)	0.62	0.20-1.89
VARC-2 bleeding	16 (7.3%)	10 (9.6%)	6 (5.2%)	0.42	0.19-0.94
VARC-2 stroke	12 (5.5%)	6 (5.8%)	6 (5.2%)	0.61	0.26-1.41
CVVH	9 (4.1%)	3 (2.9%)	6 (5.2%)	2.50	0.85-7.39
Pacemaker implantation	15 (6.8%)	7 (6.7%)	8 (7%)	1.02	0.47-2.21
PPM	47 (26%)	31 (34%)	16 (17%)	0.42	0.26-0.7

The table reports the results of the univariable weighted logistic regression models evaluating the association with valve type. Models’ results are reported as odds ratio for ME vs Intuity together with lower and upper bound of the 95% confidence interval.

Abbreviations: CVVH, continuous veno-venous hemofiltration; PPM, patient-prosthesis mismatch; VARC, Valve Academic Research Consortium.

**Table 4.** Post-Operatory Haemodynamic Characteristics

Overall	Magna Ease (n = 105)	Intuity (n = 115)	**AME (Intuity vs ME)**	**95% CI**	***P*-value**
Peak gradient (mmHg)	27 (20-35)	20 (16-25)	−6.98	−9.74 to -4.24	<.001
Mean gradient (mmHg)	16 (12-21)	12 (9-14)	−5.2	−6.83 to -3.58	<.001
AVAi (cm^2^/m^2^)	0.91 (0.77-1.05)	1.02 (0.89-1.12)	0.082	−0.01 to 0.17	.082
**Prosthesis 19**	**Magna Ease (n = 25)**	**Intuity (n = 24)**			
Peak gradient (mmHg)	34 (25-38)	28 (18-33)	−6.62	−12.37 to -0.87	.024
Mean gradient (mmHg)	20.5 (14.8-23)	17 (13.2-18)	−5.11	−8.53 to -1.68	.004
AVAi (cm^2^/m^2^)	0.96 (0.73-1.04)	0.92 (0.8-1.04)	−0.007	−0.16 to 0.14	.929
**Prosthesis 21**	**Magna Ease (n = 80)**	**Intuity (n = 91)**			
Peak gradient (mmHg)	24 (19-34)	20 (15-24)	−6.74	−9.62 to -3.86	<.001
Mean gradient (mmHg)	15 (12-20)	12 (9-13)	−4.85	−6.46 to -3.24	<.001
AVAi (cm^2^/m^2^)	0.9 (0.78-1.05)	1.03 (0.93-1.14)	0.1117	−0.0016 to 0.225	.053

Continuous data are median (I quartile-III quartile), categorical data are absolute numbers (percentages). The table reports results of the weighted Gamma models evaluating the association with valve type. Gamma models’ results are reported as average marginal effect (AME) for ME vs Intuity, lower and upper bound of the 95% confidence interval, and *P*-values.

Abbreviation: AVAi, aortic valve area indexed.

Echocardiographic results showed lower transvalvular gradients in the Intuity group. Peak gradients were 20 mmHg [IQR = 16-25] with Intuity vs 27 mmHg [IQR = 20-35] with ME (*P* < .001), while mean gradients were 12 mmHg [IQR = 9-14] vs 16 mmHg [IQR = 12-21], respectively (*P* < .001). These differences persisted across valve sizes. Interaction testing between prosthesis size and treatment group did not reveal consistent statistical significance. The *P*-values for interaction were as follows: 0.136 for postoperative peak gradient, 0.051 for postoperative mean gradient, 0.163 for postoperative aortic regurgitation, 0.016 for first follow-up peak gradient, 0.161 for first follow-up mean gradient, and 0.640 for first follow-up aortic regurgitation. PPM, defined as AVAi <0.85 cm^2^/m^2^, was observed in 26% overall but significantly less recurrent with Intuity (17% vs 34%; OR = 0.42; 95% CI, 0.26-0.70).

**Figure 2.**
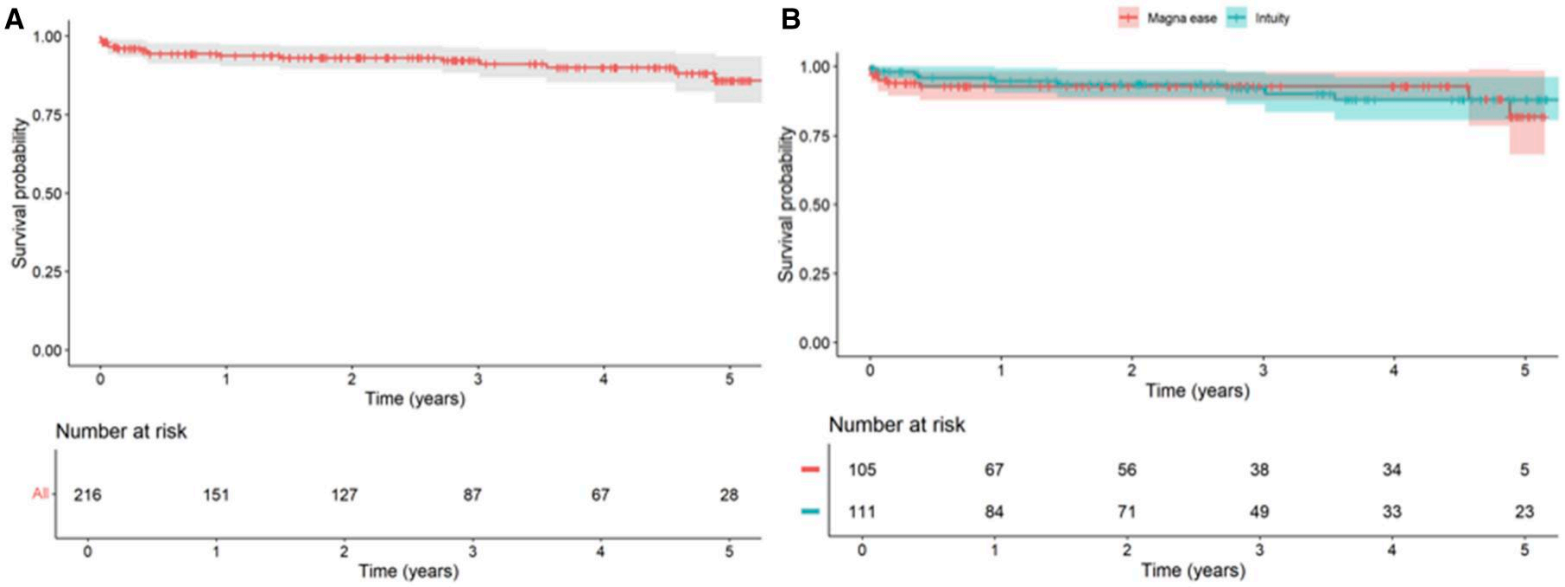
Mortality Rate During Follow-up Overall (A) and of the Two Study Devices (B)

**Figure 3.**
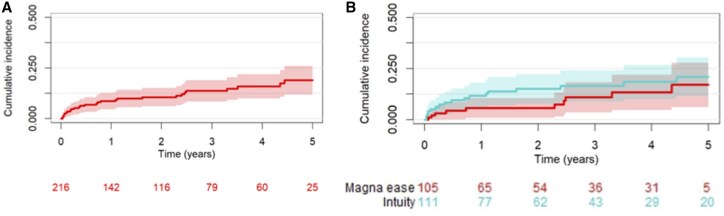
Incidence of Re-hospitalization During Follow-up Overall (A) and of the Two Study Devices (B)

Follow-up completeness reached 98%, with median duration of 755 days. Both groups had the same follow-up duration; differences in patients at risk at 5 years likely result from varying loss to follow-up rates between groups. **Table 5** shows echocardiographic parameters at 2 years, with stable peak and mean gradients overall and by valve size. **Figure 2** illustrates follow-up mortality, which was comparable between groups at 3 and 5 years. Freedom from mortality was 92% (95% CI, 86.4-98) at 3-years and 88% (95% CI, 80.5-96.1) at 5-years for Intuity vs 92.8% (95% CI, 87.8-98.1) at 3-years and 81.9% (95% CI, 68-98.5) at 5-years for ME (Cox regression *P* = .76). **Figure 3** shows rehospitalization rates, also similar: 16.4% (95% CI, 8.7-24) at 3-years and 20.9% (95% CI, 11.4-30.4) at 5-years for Intuity vs 10.9% (95% CI, 3.5-18.2) at 3-years and 17% (95% CI, 6.2-27.8) at 5-years for ME (*P* = .57). Although 115 patients received an Intuity valve size 19 or 21, follow-up data were not available for all individuals; therefore, the number at risk shown in **Figures 2 and 3** reflects only those with complete follow-up information.

**Table 5.** Follow-up Haemodynamic Characteristics

Overall	Patients number	Magna Ease	Intuity	**AME (Intuity vs ME)**	95% CI	***P*-value**
2y FU Peak gradient (mmHg)	103	28 (21-36)	20 (16-24)	−7.13	−11.59 to -2.67	.002
2y FU Mean gradient (mmHg)	99	15.5 (12-19.2)	12 (9-13.5)	−3.63	−0.41 to 0.14	.006
**Prosthesis 19**		**Magna Ease**	**Intuity**			
2 y FU Peak gradient (mmHg)	20	34 (26-40)	32 (29-38)	−0.38	−11.37 to 10.61	.946
2y FU Mean gradient (mmHg)	22	18.5 (16-23.5)	17.5 (13-19.8)	−2.29	−8.46 to 3.88	.466
**Prosthesis 21**		**Magna Ease**	**Intuity**			
2y FU Peak gradient (mmHg)	83	25 (20-32)	18 (16-22)	−8.43	−12.6 to -4.26	<.001
2y FU Mean gradient (mmHg)	77	14 (10.8-18.2)	11 (9-12)	−3.51	−5.92 to -1.098	.004

Continuous data are median (I quartile-III quartile), categorical data are absolute numbers (percentages). The table reports results of the weighted Gamma models evaluating the association with valve type. Gamma models’ results are reported as average marginal effect (AME) for ME vs Intuity, lower and upper bound of the 95% confidence interval, and *P*-values.

Abbreviations: FU, follow-up; 2y, 2-years.

## DISCUSSION

The central results of this study are that in small annuli, Intuity provides better haemodynamic outcomes (both mean and peak gradients) compared to ME in 30-days evaluation and also at 5-years follow-up. Moreover, the post-operative outcomes are similar between the 2 devices, including PPI. Similarly, no significant differences were observed in terms of mid-term survival or rates of rehospitalization. Despite identical labelled valve sizes, the Intuity prosthesis showed enhanced haemodynamic performance, likely due to its specific design features. In particular, the incorporation of a subannular skirt facilitates improved expansion and anchoring within the left ventricular outflow tract. While the leaflet structure and geometric orifice area are the same in both valves, the subannular deployment of the Intuity may optimize flow dynamics, resulting in lower transvalvular gradients and a larger effective orifice area. In a previous study by our group,^19^ we found that the RD bioprostheses outperformed the ME across all sizes, with lower mean and peak gradients and a larger AVAi. These findings were supported by Rahmanian et al,^20^ who conducted a propensity score-matched analysis of 163 patients receiving either the RD or ME. Their results confirmed significantly better haemodynamic performance in the RD group. The statistically and clinically significant average marginal effect observed in favour of the Intuity prosthesis supports its haemodynamic benefit in small annuli, where the risk of PPM is intrinsically higher. These results may inform surgical decision-making when selecting the most appropriate prostheses in challenging anatomical scenarios.^21^^,^^22^ As shown in **Tables 4 and 5**, the superiority of the Intuity is evident both at 30 days (**Table 4**) and at long-term follow-up (**Table 5**).

Arribas-Leal et al^23^ also reported good clinical and haemodynamic outcomes with the RD in small annuli, although differences in study design and methodology limit direct comparisons. Chiariello et al^24^ found that, in elderly patients (>75 years-old) with annuli <21 mm, the Intuity was safe, effective, and associated with lower transvalvular gradients, comparable effective orifice area, and favourable clinical outcomes. It is important to note that these comparisons are intended to highlight the haemodynamic performance of the Intuity, without addressing the overall performance of other bioprostheses. PPM remains a relevant clinical concern, particularly in patients receiving small-sized prostheses. In our study, PPM was observed in 26% of the overall cohort. Particularly, its incidence was significantly lower in the Intuity group compared to the ME group (17% vs 34%), suggesting a haemodynamic benefit of the RD prostheses even in patients with small annular anatomy. These findings are consistent with previous studies emphasizing the importance of minimizing PPM, which has been associated with worse long-term outcomes, including increased mortality and impaired functional recovery. In an independent study from our group,^25^ we examined the discrepancy between predicted and observed PPM and highlighted the importance of individualized prosthesis selection to prevent underestimation of mismatch risk. Our current data support the concept that prosthesis choice, beyond annulus size alone, plays a key role in reducing the incidence of PPM.

No significant differences were observed in early or mid-term complications between groups, and mid-term survival was comparable. Interestingly, the observed 30-day mortality, based on VARC-2 criteria, was higher than the risk predicted by EuroSCORE II. This discrepancy may reflect limitations of EuroSCORE II in accounting for anatomical or procedural variables such as small annuli or RD prostheses, underscoring the need for more tailored risk models. While the clinical impact of a few mmHg differences in transvalvular gradient may seem minimal, such differences could accelerate SVD over time.^26^ This is particularly relevant for long-term follow-up, even in patients with preserved left ventricular function.

### Limitations

This study has several limitations, primarily due to its retrospective design, which may introduce inherent biases. Another limitation is the lack of a universally accepted definition for a small aortic annulus. Although we followed a commonly used definition from the literature, this may not fully account for patient-specific anatomical variations, and results should therefore be interpreted cautiously. Additionally, the choice of prosthesis was left to the discretion of the surgeon, introducing the possibility of selection bias. The relatively small sample size may have reduced the statistical power to detect significant differences, especially in exploratory subgroup analyses based on ACC and CPB times. These subgroup findings, although potentially clinically relevant, should be considered hypothesis-generating rather than definitive. Future studies with larger cohorts are necessary to validate and expand on these observations. Thirty-day echocardiographic assessments were standardized using the same equipment and laboratory; however, follow-up at various centres may introduce measurement variability. Long-term data are needed to evaluate durability, SVD, and late outcomes. A broader comparison including all valve types and surgical techniques would better clarify their relative advantages and limitations.

## CONCLUSIONS

In conclusion, in patients with small aortic annuli, RD prostheses offer superior haemodynamic performance and shorter CPB and ACC times compared to stented prostheses during SAVR, while early outcomes, mid-term survival and rehospitalization rates remain comparable between devices.

## Supplementary Material

ivaf241_Supplementary_Data

## Data Availability

The data underlying this article are available in the article and in its online supplementary material.

## References

[1] Dahlbacka S , Laakso T, Kinnunen EM, et al Patient-prosthesis mismatch worsens long-term survival: insights from the FinnValve registry. Ann Thorac Surg. 2021;111:1284-1290. 10.1016/j.athoracsur.2020.06.026 32805269

[2] Shih E , DiMaio JM, Squiers JJ, et al Outcomes of aortic root enlargement during isolated aortic valve replacement. J Card Surg. 2022;37:2389-2394. 10.1111/jocs.16645 35598292

[3] Bening C , Hamouda K, Oezkur M, et al Rapid deployment valve system shortens operative times for aortic valve replacement through right anterior minithoracotomy. J Cardiothorac Surg. 2017;12:27. 28511707 10.1186/s13019-017-0598-0 PMC5434633

[4] Ai L , Chen H, Lin V, Bapat VN. Rapid deployment aortic valves deliver superior hemodynamic performance *in vitro*. Innovations (Phila). 2017;12:338-345. 29023351 10.1097/IMI.0000000000000407 PMC5657464

[5] Freitas-Ferraz AB , Tirado-Conte G, Dagenais F, et al Aortic stenosis and small aortic annulus. Circulation. 2019;139:2685-2702. 10.1161/CIRCULATIONAHA.118.038408 31157994

[6] Repossini A , Di Bacco L, Passaretti B, et al Early hemodynamics and clinical outcomes of isolated aortic valve replacement with stentless or transcatheter valve in intermediate-risk patients. J Thorac Cardiovasc Surg. 2017;153:549-558.e3. 10.1016/j.jtcvs.2016.10.086 27939031

[7] D'Onofrio A , Salizzoni S, Filippini C, et al Surgical aortic valve replacement with new-generation bioprostheses: sutureless versus rapid-deployment. J Thorac Cardiovasc Surg. 2020;159:432-442.e1. 31213376 10.1016/j.jtcvs.2019.02.135

[8] Nashef SAM , Roques F, Sharples LD, et al EuroSCORE II. Eur J Cardiothorac Surg. 2012;41:734-744. 22378855 10.1093/ejcts/ezs043

[9] Kappetein AP , Head SJ, Généreux P, et al; Valve Academic Research Consortium (VARC)-2. Updated standardized endpoint definitions for transcatheter aortic valve implantation: the valve academic research consortium-2 consensus document (VARC-2). Eur J Cardiothorac Surg. 2012;42:S45-S60. 10.1093/ejcts/ezs533 23026738

[10] Borger MA , Dohmen P, Misfeld M, Mohr FW. Minimal invasive implantation of an EDWARDS INTUITY rapid deployment aortic valve. Multimed Man Cardiothorac Surg. 2013;2013:mmt011. 10.1093/mmcts/mmt011 24413009

[11] Glauber M , Miceli A, Di Bacco L. Sutureless and rapid deployment valves: implantation technique from a to Z-the INTUITY elite valve. Ann Cardiothorac Surg. 2020;9:417-423. 33102182 10.21037/acs-2020-surd-23-intuity PMC7548214

[12] Imai K , Ratkovic M. Covariate balancing propensity score. J R Stat Soc Ser B Stat Methodol. 2014;76:243-263.

[13] Austin PC. An introduction to propensity score methods for reducing the effects of confounding in observational studies. Multivariate Behav Res. 2011;46:399-424. 10.1080/00273171.2011.568786 21818162 PMC3144483

[14] Austin PC , Stuart EA. Moving towards best practice when using inverse probability of treatment weighting (IPTW) using the propensity score to estimate causal treatment effects in observational studies. Stat Med. 2015;34:3661-3679. 10.1002/sim.6607 26238958 PMC4626409

[15] Austin PC. Propensity-score matching in the cardiovascular surgery literature from 2004 to 2006: a systematic review and suggestions for improvement. J Thorac Cardiovasc Surg. 2007;134:1128-1135. 10.1016/j.jtcvs.2007.07.021 17976439

[16] Greifer N. WeightIt: weighting for covariate balance in observational studies. 2024 [cited 2024 Feb 22]. Available from: https://cran.r-project.org/ web/packages/WeightIt/index.html.

[17] Fong C , Ratkovic M, Imai K. CBPS: covariate balancing propensity score. 2019. Available from: https://CRAN.R-project.org/package=CBPS

[18] Leeper TJ. *margins: Marginal Effects for Model Objects.* 2021. Available from: https://cran.r-project.org/web/packages/margins/index.html

[19] D'Onofrio A , Cibin G, Lorenzoni G, et al Propensity-weighted comparison of conventional stented and rapid-deployment aortic bioprostheses. Curr Probl Cardiol. 2023;48:101426. 10.1016/j.cpcardiol.2022.101426 36181783

[20] Rahmanian PB , Kaya S, Eghbalzadeh K, Menghesha H, Madershahian N, Wahlers T. Rapid deployment aortic valve replacement: excellent results and increased effective orifice areas. Ann Thorac Surg. 2018;105:24-30. 29132703 10.1016/j.athoracsur.2017.07.047

[21] Elmahdy W , Osman M, Farag M, et al Prosthesis-patient mismatch increases early and late mortality in low risk aortic valve replacement. Semin Thorac Cardiovasc Surg. 2021;33:23-30. 32439547 10.1053/j.semtcvs.2020.05.006

[22] Pibarot P , Weissman NJ, Stewart WJ, et al Incidence and sequelae of prosthesis-patient mismatch in transcatheter versus surgical valve replacement in high-risk patients with severe aortic stenosis: a PARTNER trial cohort—a analysis. J Am Coll Cardiol. 2014;64:1323-1334. 25257633 10.1016/j.jacc.2014.06.1195 PMC4237285

[23] Arribas-Leal JM , Rivera-Caravaca JM, Aranda-Domene R, et al Mid-term outcomes of rapid deployment aortic prostheses in patients with small aortic annulus. Interact CardioVasc Thorac Surg. 2021;33:695-701. 10.1093/icvts/ivab175 34179967 PMC8923414

[24] Chiariello GA , Bruno P, Villa E, et al Aortic valve replacement in elderly patients with small aortic annulus: results with three different bioprostheses. Innovations (Phila). 2019;14:27-36. 10.1177/1556984519826430 30848711

[25] Cibin G , D'Onofrio A, Lorenzoni G, et al Predicted vs. observed prosthesis-patient mismatch after surgical aortic valve replacement. Medicina (Kaunas). 2025;61:743. 10.3390/medicina61040743 40283034 PMC12028624

[26] Mohty D , Dumesnil JG, Echahidi N, et al Impact of prosthesis-patient mismatch on long-term survival after aortic valve replacement: influence of age, obesity, and left ventricular dysfunction. J Am Coll Cardiol. 2009;53:39-47. 10.1016/j.jacc.2008.09.022 19118723

