# Supplementary figures and images for: Propensity score analysis of stented versus rapid deployment aortic bioprostheses in patients with small aortic annulus
Source: Interdiscip Cardiovasc Thorac Surg. 2025 Oct 10;40(11):ivaf241. doi: 10.1093/icvts/ivaf241 (PMC12596474; doi:10.1093/icvts/ivaf241)

### Covariate Balance

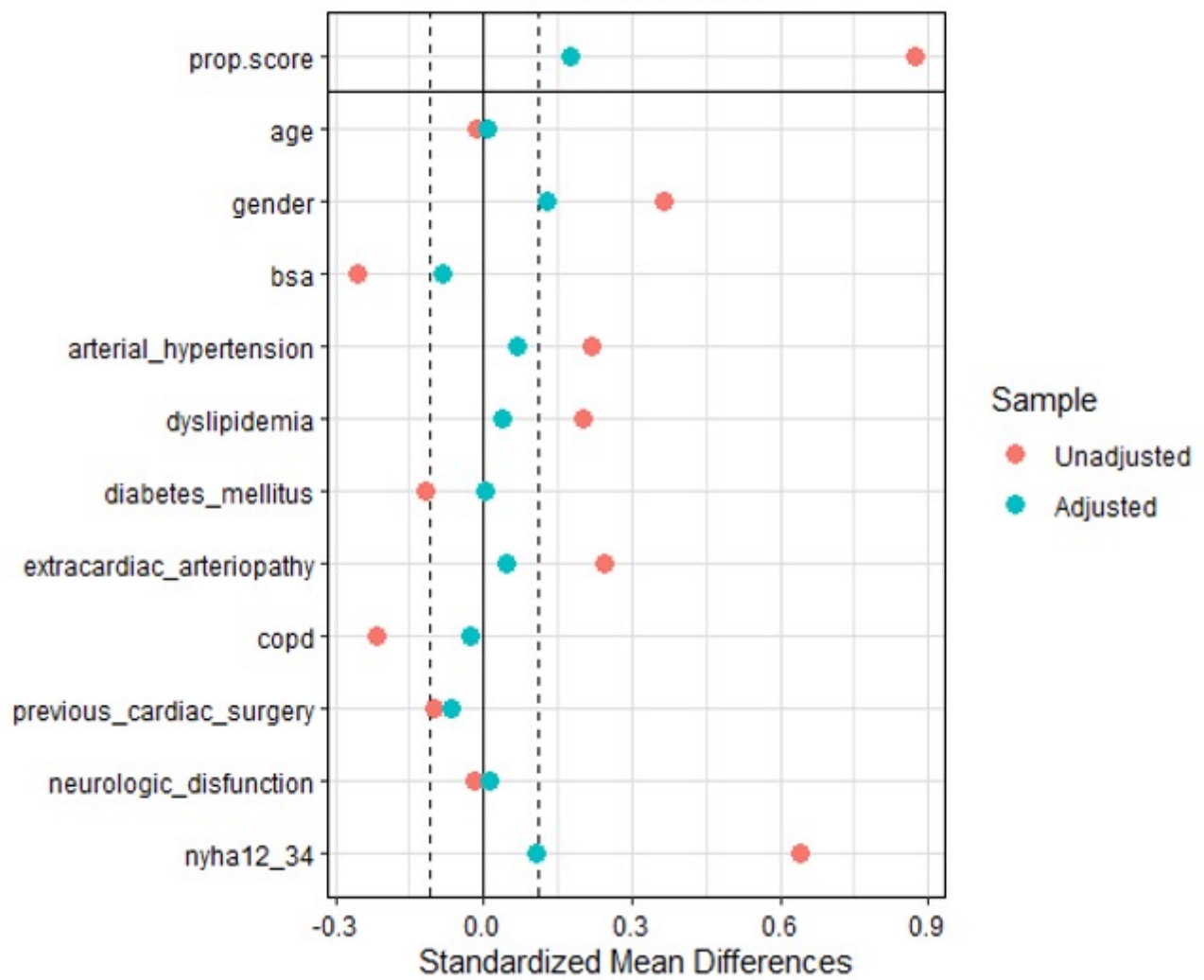

Supplement: ivaf241_Supplementary_Data [file ivaf241_supplementary_data.zip › Fig S1.pdf]
